# ENL mutation and AML: a new model that reveals oncogenic condensate's function in leukemogenesis

**DOI:** 10.1002/1878-0261.13731

**Published:** 2024-09-26

**Authors:** Zhong Fan, Yanan Jiang, Xiaotian Zhang

**Affiliations:** ^1^ Department of Biochemistry and Molecular Biology University of Texas Health Science Center at Houston, McGovern Medical School TX USA; ^2^ Department of Pediatrics The First Affiliated Hospital, Sun Yat‐sen University Guangzhou China

**Keywords:** ENL YEATS, histone modifications, TDI‐11055, transcriptional condensates, tumorigenesis

## Abstract

Precise regulation of gene expression is essential for proper development and the maintenance of homeostasis in organisms. Studies have shown that some transcriptional regulatory proteins influence gene expression through the formation of dynamic, locally concentrated assemblies known as condensates, while dysregulation of transcriptional condensates was associated with several cancers, such as Ewing sarcoma and AML [Wang Y et al. (2023) *Nat Chem Biol* 19, 1223–1234; Chandra B et al. (2022) *Cancer Discov* 12, 1152–1169]. Mutations in the histone acetylation “reader” eleven‐nineteen‐leukemia (ENL) have been shown to form discrete condensates at endogenous genomic targets, but it remains unclear how ENL mutations drive tumorigenesis and whether it is correlated with their condensate formation property. Liu et al. now show, using a conditional knock‐in mouse model, that ENL YEATS domain mutation is a *bona fide* oncogenic driver for AML. This mutant ENL forms condensates in hematopoietic stem/progenitor cells at the genomic loci of key leukemogenic genes, including *Meis1* and *Hoxa* cluster genes, and disrupting condensate formation via mutagenesis impairs its chromatin and oncogenic function. Furthermore, they show that small‐molecule inhibition of the acetyl‐binding activity displaces ENL mutant condensates from oncogenic target loci, and this inhibitor significantly impairs the onset and progression of AML driven by mutant ENL *in vivo*.

## Graphical abstract

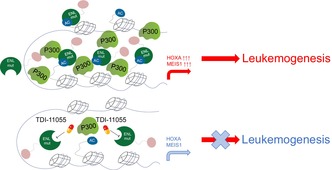

**Abbreviations**
AHD: ANC1 homology domain
AML: acute myeloid leukemia
ENL: eleven‐nineteen lysine‐rich leukemia
GMP: granulocyte/monocyte progenitors
GO: gene ontology
HSC: hematopoietic stem cells
IDR: intrinsically disordered region
MLL: mixed‐lineage leukemia
TLRs: toll‐like receptors
YEATS: Yaf9, ENL, AF9, Taf14, Sas5

## 1 ENL and mutant ENL in AML

Eleven‐nineteen‐leukemia (ENL), encoded by the MLLT1 gene, belongs to a protein family that contains a well‐conserved YEATS domain, which can recognize histone lysine acylation, as an epigenetic reader [1, 2]. This recognition facilitates the recruitment of cofactors such as the super elongation complex (SEC/P‐TEFb) and DOT1L, promoting efficient transcriptional elongation [1, 2]. Studies have shown that ENL plays a critical role in cancer through various mechanisms. It frequently undergoes chromosomal translocation with mixed‐lineage leukemia protein (MLL1 or KMT2A), resulting in the formation of the potent oncogenic MLL‐ENL fusion protein, which is associated with poor prognosis [3].

Moreover, the wild‐type (WT) ENL is required for the maintenance of oncogenic programs in subsets of AML [1, 2]. In addition to WT ENL, hotspot mutations frequently occur in the ENL YEATS domain in patients with Wilms tumor [4]. Wan et al. [5] also discovered that these mutations enhanced ENL self‐association, recruiting more ENL and forming discrete and dynamic nuclear puncta, thereby regulating gene transcription and disrupting the normal control of cell fate during development. Furthermore, the authors showed that ENL mutations induced abnormal condensate formation at specific genomic sites through dose‐dependent multivalent homotypic and heterotypic interactions. Disrupting these interactions by restoring the β8 length to WT levels, expressing deletion variants of other ENL domain (IDR, AHD), or disrupting the interactions between ENL and SEC/DOT1L would interfere with condensate formation, leading to impaired oncogenic gene activation. In addition, markedly overexpressed ENL mutants could form large, non‐chromatin‐associated condensates that fail to activate transcription [6].

Building on these findings, they presented an oral available small‐molecule inhibitor targeting ENL‐TDI‐11055. It disrupted ENL's association with chromatin by inhibiting its YEATS domain binding to acylated histones. This compound demonstrated strong efficacy in preclinical models of acute myeloid leukemia (AML) carrying MLL rearrangements and NPM1 mutations [7]. However, how the mutant ENL acts to promote leukemogenesis in unknown. Particularly, whether condensates of ENL contribute to AML development is unknown.

## 2 The link between ENL mutations, condensate formation, and tumorigenesis

Recently, Liu et al. published an article titled “Condensate‐promoting ENL mutation drives tumorigenesis *in vivo* through dynamic regulation of histone modifications and gene expression” in *Cancer Discovery* [8]. This study revealed the molecular mechanism by which ENL mutations induce acute myeloid leukemia and showed that their oncogenic function relied on the formation of abnormal transcriptional condensates. By inhibiting the formation of ENL condensates at target genes (*Hoxa* cluster and *Meis1* gene) with small‐molecule TDI‐11055, the gene dysregulation and cancer development caused by ENL mutations got effectively repressed.

First, the authors created a conditional knock‐in mouse model for the most prevalent ENL YEATS domain mutation found in cancer, referred to as ENL‐T1. The *in vivo* studies indicate that heterozygous expression of the ENL‐T1 mutant drives the development of aggressive acute leukemia. In addition, they performed targeted genomic DNA sequencing on a panel of 611 cancer‐related genes to identify comutated genes with ENL. In addition to the predominant heterozygous Enl‐T1 mutation observed in nearly all cells, only two other mutations were found in a minority of the cells: one in the Kit gene and one in the *Ptpn11* gene (p.E76K, VAF ~ 0.1). The *Kit* mutation is not currently associated with leukemogenesis. While the Ptpn11 mutation has been linked to leukemia development in mouse models, it results in less severe phenotypes compared with the *Enl‐T1* mutation in the same *Mx1‐cre* model. These suggest that the *Enl‐T1* mutation is sufficient to drive AML development.

Further investigation revealed that mutant ENL disrupts normal hematopoiesis, results in a decrease of hematopoietic stem cells (HSCs) and most committed progenitors while inducing the expansion of granulocyte/monocyte progenitors (GMP) and abnormal committed myeloid progenitors (cKit+Mac1+), ultimately resulting in AML. By performing RNA sequencing on LSK and GMP cells from preleukemic Enl‐T1 mice, as well as leukemic GMP (L‐GMP), cKit+Mac1+, and cKit−Mac1+ myeloid cells from leukemic Enl‐T1 mice, they found that compared with WT mice, the presence of Enl‐T1 led to significantly higher *Hoxa* gene expression in LSK cells, which were aberrantly sustained in progenitor cells (such as GMP, L‐GMP, cKit+Mac1+) and in terminally differentiated myeloid cells (cKit−Mac1+). Further gene ontology (GO) term analysis revealed that gene signatures associated with inflammatory and immune pathways were significantly enriched among Enl‐T1‐upregulated genes in LSK, GMP, and L‐GMP cells. These signatures include genes related to inflammation and are known to impact the bone marrow microenvironment during leukemogenesis, such as those encoding Toll‐like receptors (TLRs) and interleukins.

The authors then questioned whether changes in the chromatin state result in the observed transcriptional alterations. First, they profiled genome‐wide distribution of histone 3 lysine 27 acetylation (H3K27ac), via CUT&Tag in Enl‐WT and Enl‐T1 LSK, GMP, L‐GMP, cKit+Mac1+, and cKit−Mac1+ cells between WT and T1 mice. They uncovered that mutant ENL recruited the acetyltransferase p300, leading to increased levels of H3K27ac in hematopoietic cells, thereby modulating the expression of key leukemia genes such as *Hoxa* cluster genes and *Meis1*. Given that Polycomb‐mediated methylation of H3K27 (H3K27me3) also plays important roles in development, they also conducted H3K27me3 CUT&Tag analyses in the aforementioned cell populations. They found that in cKit+Mac1+ and cKit−Mac1+ cells, Enl‐T1 caused a significant reduction in signal intensity at certain broad H3K27me3 peaks that are functionally associated with developmental pathways. In summary, their epigenomic analyses reveal that Enl‐T1 mice exhibit dynamic changes in both active and repressive histone modifications, which are linked to altered expression of specific development‐related genes.

As mentioned before, mutant ENL could form abnormal condensates in cell lines. The researchers next investigated whether this phenomenon occurs in primary hematopoietic cells and if it is associated with leukemia progression. By using immunofluorescence DNA‐FISH, they expressed various ENL variants in hematopoietic stem cells and compared the differences. The results showed that ENL cancer mutants form prominent condensates, primarily at *Hoxa* cluster genes and *Meis1*. Introducing specific point mutations to ENL cancer mutants could inhibit the formation of condensates and suppress their epigenetic regulation and oncogenic functions. This indicated that condensates driven by ENL cancer mutations are closely related to the cancer development they mediate.

**Fig. 1:**
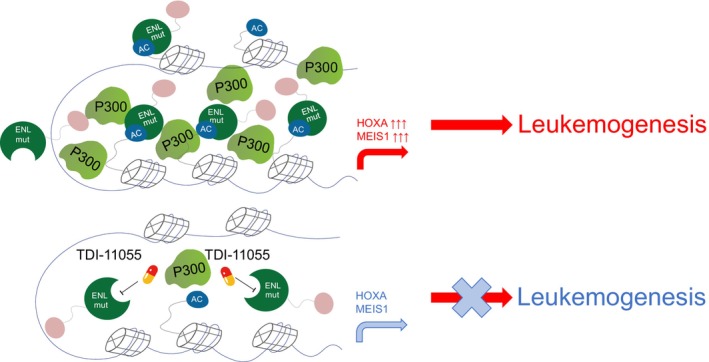
The eleven‐nineteen‐leukemia (ENL) mutant forms condensates at specific gene loci, leading to alterations in transcription and histone modifications in hematopoietic stem and progenitor cells (HSPC), thereby driving leukemogenesis. TDI‐11055 displaces ENL mutant condensates from these target loci, diminishes their impact on histone modifications and gene activation, and effectively disrupts leukemogenesis.

Then, how can these oncogenic condensates be targeted? Similar to WT ENL, mutant ENL relies on its YEATS domain to recognize histone acetylation. In this study, they found that the small‐molecule TDI‐11055 disrupted the association of ENL mutant condensates with their target genes, while also inhibiting p300/H3K27ac elevation and the aberrant activation of target genes mediated by these condensates (Fig. 1). Animal experiments further demonstrated that this small‐molecule can inhibit ENL mutation‐driven leukemia, highlighting the potential of targeting transcriptional condensates in cancer therapy.

This work demonstrates that ENL mutations can induce leukemia by forming abnormal condensates, thereby expanding our understanding of the pathogenesis of leukemia. Additionally, this study provides strong evidence that dysregulation of transcriptional condensates plays a significant role in cancer development, opening new avenues for research into the molecular mechanisms of the disease. Furthermore, the paper shows how small‐molecule inhibitors targeting histone “readers” can effectively suppress leukemia by interfering with the positioning of these condensates on target genes. The study provides strong evidence for the role of transcriptional condensates in disease and highlights the potential of targeting these structures in cancer therapy.

However, several key questions remain to be explored. First, why do ENL mutants specifically target the HOXA/MEIS1 loci? Further research should focus on elucidating the mechanisms underlying ENL's specific regulation of Hoxa and Meis1 in both hematopoiesis and kidney development, including the role of other interacting partner proteins beyond EP300. This includes investigating the interactions between ENL and transcription factors, or RNA that may contribute to the specific targeting of ENL‐T1. Although this study successfully constructed a model to elucidate ENL‐driven leukemogenesis, it is worth noting that ENL mutations also co‐occur with other mutations in human AML. Investigating the comutation genes associated with ENL mutations will better aid in the development of targeted combination therapies, potentially improving the prognosis of AML. This study has demonstrated that ENL condensates are closely related to its chromatin binding affinity. Further research is needed to clarify whether this mechanism applies to other subtypes of AML carrying mutations that also lead to oncogenic condensate changes [9, 10]. Addressing these questions could provide deeper insights into the molecular basis of AML and refine therapeutic strategies targeting transcriptional condensates in cancer.

## Conflict of interest

The authors declare no conflict of interest.

## Author contributions

XZ and ZF conceived and designed the project; ZF and YJ wrote the paper; XZ revising it critically for important intellectual content. All authors have read and approved the final manuscript.
